# A novel miR-219-SMC4-JAK2/Stat3 regulatory pathway in human hepatocellular carcinoma

**DOI:** 10.1186/1756-9966-33-55

**Published:** 2014-06-30

**Authors:** Bo Zhou, Hongxu Chen, Dong Wei, Yi Kuang, Xiaobiao Zhao, Guangyao Li, Jun Xie, Ping Chen

**Affiliations:** 1 Department of Hepatobiliary Surgery, Daping Hospital and Research Institute of Surgery, The Third Military Medical University, Chongqing, China

**Keywords:** SMC4, Hepatocellular carcinoma, miR-219, JAK2/Stat3

## Abstract

**Background:**

To understand the involvement of structural maintenance of chromosome 4 (SMC4) in the development and progression of hepatocellular carcinoma (HCC).

**Methods:**

Real-time quantitative PCR and Western Blotting were applied to measure the expression of SMC4 in HCC samples and cell lines. The tumor-promoting effect of SMC4 was determined by WST-1, soft agar colony formation, cell motility and invasion assays. The SMC4 target signal pathway was identified by luciferase reporter and real-time quantitative PCR assays.

**Results:**

The upregulation of SMC4 was frequently detected in HCC samples and cell lines. Functional assays demonstrated that SMC4 could effectively promote tumor cell growth rate, colony formation in soft agar, wound-healing and invasion. Further studies showed that increased miR-219 levels caused a significant decrease in the SMC4 expression, and SMC4 inhibitor downregulated JAK2/Stat3 expression at both the mRNA and protein levels.

**Conclusions:**

Our findings provide new insight into SMC4 function and the mechanisms of growth and invasion of HCC.

## Introduction

Hepatocellular carcinoma (HCC) is the most common primary liver cancer, and the third most deadly type of cancer globally, following lung and stomach cancers [1]. With more than 750,000 new cases diagnosed every year worldwide, HCC is the sixth most common neoplasm [2,3]. There is considerable controversy about the role of surveillance in its management [4]. Small HCCs are amenable to more effective treatment [5,6]. However, HCC is diagnosed at advanced stages in most patients when only limited therapeutic options are available.

From a molecular perspective, HCC is a highly heterogeneous tumor [7]. Increasing evidence suggests that its complexity and clinical variability mainly depend on various molecular alterations that arise during the development and progression of this disease [7-10]. Previous studies have reported on aberrant activation of various cellular pathways such as GSK-3 beta-C/EBP alpha-miR-122-Insulin Growth Factor 1 Receptor [11], HDGF-related protein-3 [12], FoxM1/ACP5 [13], JAK/STAT [14], miR-219-5p/glypican-3 [15] etc. Moreover, recent studies have demonstrated the importance of the JAK/STAT pathway in the development of HCC, and suggest that the use of JAK/STAT inhibitors might have potential in the treatment of HCC [16]. miR-219 has been shown to exert tumor-suppressive effects during hepatic carcinogenesis through negative regulation of GPC3 expression [15]. Therefore, identification of the predominant molecular mechanisms as therapeutic options for HCC is a research area of great importance and interest.

In a previous study, we demonstrated that structural maintenance of chromosome 4 (SMC4) is highly expressed in human HCC tissues and cell lines. We further showed that expression of SMC4 may be useful for the early detection of HCC, and is related to the progression and invasion the tumor [17]. In the current study, we aimed to provide new insight into SMC4 function, and the mechanisms of growth and invasion of HCC.

## Materials and Methods

### Cell lines and clinical samples

Human hepatocellular carcinoma 97-H, HepG2, Bel-7405, and smmc-7721 cell lines and human normal liver L02(also called HL-7702) cell line ,were purchased from Chinese Academy of Sciences (Shanghai, China). A total of 72 HCC samples from primary tumor, paracancerous samples and adjacent non-tumor sites were obtained from patients with primary liver cancer prior to any therapy. Written informed consent from all patients was obtained prior to participation in the study. The adjacent non-tumor area was subsequently verified by histology to be free of tumor infiltration. Clinical samples were obtained from Department of Hepatobiliary Surgery, Daping Hospital and Research Institute of Surgery, the Third Military Medical University between September 2009 and December 2010. All samples were immediately snap-frozen and kept at −80°C until use. This study was approved by the Ethics Committee of Daping Hospital and Research Institute of Surgery, the Third Military Medical University, Chongqing, China. (#ChiCTR- DDT-11001845).

### Real-time quantitative PCR

Total RNA was extracted from cultured cells or frozen tissues using TRIzol reagent (Invitrogen, Carlsbad, California, USA), and RNA was reverse-transcribed by an RT-PCR kit (Invitrogen, Carlsbad, California, USA) according to the manufacturer’s instructions. Quantitative PCR was then carried out with primers for miR-219, SMC4, JAK2, and Stat3 with SYBR Green PCR Master Mix (Invitrogen, Carlsbad, California, USA) in a real-time PCR System (Applied Biosystems, Carlsbad, California, USA) following standard quantitative PCR procedure [18]. Primers are shown in Table 1. SMC4, JAK2, and Stat3 levels were analyzed. The programs were as follows: PCR mixtures were denatured at 95°C for 5 min, followed by 40 cycles of 94°C for 20 s, 61°C for 20 s, and 72°C for 20 s, and a final extension at 72°C for 5 min, 55°C for 10 s. miR-219 was analyzed as follows: PCR mixtures were incubated at 95°C for 5 min, followed by 40 cycles with incubation at 95°C for 10 s, 60°C for 20 s, 72°C for 10 s. Data were normalized to the geometric mean of the housekeeping gene, β-actin and U6 (Sangon Biotech, Shanghai, China) as the control.

**Table 1:** Primer sequences used qRT-PCR

	**Forward primer**	**Reverse primer**
SMC4	5'-GAGAAAATTCTGGGACCTTT-3'	5'-TCTGAATGTCCTTGTGTTCA-3'
β-actin	5'-CATTAAGGAGAAGCTGTGCT-3'	5'-GTTGAAGGTAGTTTCGTGGA-3'
mir-219	5'- TGATTGTCCAAACGCA -3'	5'- TTTGGCACTAGCACATT -3'
U6	5'- CTCGCTTCGGCAGCACA -3'	5'- AACGCTTCACGAATTTGCGT -3'
JAK2	5'- AGCCTATCGGCATGGAATATCT -3'	5'- TAACACTGCCATCCCAAGACA -3'
STAT3	5'- ACCAGCAGTATAGCCGCTTC -3'	5'- GCCACAATCCGGGCAATCT -3'
β-actin	5'- CATTAAGGAGAAGCTGTGCT-3'	5'- GTTGAAGGTAGTTTCGTGGA -3'

### Western Blotting

Total protein was extracted from cultured cells or frozen tissues using total protein extraction kit (ProMab Biotechnologies, Richmond, USA) according to the manufacturer’s instructions. Protein extracts were separated on 10% SDS-PAGE gels and electrophoretically transferred to nitrocellulose membranes at 300 mA for 70 min. The membranes were incubated with the following antibodies and dilutions: the rabbit anti-SMC4, rabbit anti -JAk2 and rabbit anti-Stat3 at dilutions of 1:500 (Abcam, Cambridge, UK), and goat anti rabbit secondary antibody at a dilution of 1:4000 (Abcam, Cambridge, UK). The nitrocellulose membranes were exposed to X-ray films to detect the positive protein band. GAPDH (Santa Cruz Biotechnologies, CA, USA) was used as the control.

### Construction of vector and transfection

To knockdown SMC4 expression, we first designed and synthesized 3 pairs of siRNA fragments (GenePharma, Shanghai, China), and one pair of a negative control (GenePharma, Shanghai, China). Previous studies have shown that SMC4-Homo-830 (Table 2) greatly decreased SMC4 expression [17]. SMC4-Homo-830 was cloned into Pgpu/GFP/ Neo-shNC vector (GenePharma, Shanghai, China), and transfected into 97-H and HepG2, HCC cell lines. MiR-219 mimics and miR-219 inhibitor were purchased from Shanghai GenePharma Co., Ltd (Table 3). SMC4-Homo-830, miR-219 mimics and miR-219 inhibitor were then stably and transiently transfected into 97-H and HepG2 cells using Lipofectamine 2000 (Invitrogen).

**Table 2:** The sequence of homo-830

	**DNA Sequence**
Homo-830	5'-GGCCUGCAGAGAUAAUACUTT-3'
	5'-AGUAUUAUCUCUGCAGGCCTT-3'

**Table 3:** The sequences of miR-219 inhibitor and mimics

	**Sequences**
miR-219-inhibitor	5'- AGAAUUGCGUUUGGACAAUCA-3'
miR-219-mimics	5'- UGAUUGUCCAAACGCAAUUCU AAUUGCGUUUGGACAAUCAUU-3'

### Cell motility and invasion assay

Wound-healing assay and invasion assay were performed as described previously [19]. For wound healing assay, the cell layer was wounded using a sterile micropipette tip. The spread of wound closure was observed for 72 h after transfection, and photographed under a microscope. Invasion assays were performed with a chamber containing a polycarbonate membrane (8 μm pore size) and coated with a layer of extracellular matrix (Beyotime Institute of Biotechnology, Shanghai, China) according to the manufacturer's instructions. After 72 h of transfection, the number of cells that penetrated the matrigel was counted from 5 randomly selected fields (left, right, up, down and middle).

### WST-1 and soft agar colony formation assays

For WST-1 assays, cells were plated in 96-well culture plates (0.1-5 × 10^4^/ml), and incubated for 0–72 h after transfection with 10 μl WST-1/ECS. The wells with WST-1/ECS (Beyotime Institute of Biotechnology, Shanghai, China), and without cells as the blank control were incubated for 2 h. The wells were measured at 450 nm. Experiments were repeated three times independently. For colony formation assays, 0.3% of the superstratum agar, 0.6% low melting point agarose and cell cultures were mixed in equal volumes. To each well was added 1 ml of superstratum agar and 100 μl single cell suspension(about 1000 cell/well), and incubated for 2 weeks at 37°C and 5% CO_2_.

### Luciferase Reporter Assay

Luciferase reporter assays were used to characterize the miR-219 promoter and SMC4 -targeted 3’-UTR activity. Stable miR-219 mimics and miR-219 inhibitor (97-H and HepG2) were transfected with the reporter constructs (Additional file 1). The luciferase activity was measured by a luciferin enzyme detection assay kit (Promega, Wisconsin, USA).

### Co-immunoprecipitation

97-H and HepG2 cells were collected, and total protein was extracted in RIPA buffer. Cell lysates were centrifuged and the pellets discarded. Protein lysates were incubated with 2 μg Stat3 antibody (1:150) overnight followed by 20 μl Protein A + G Agarose and incubated for 2 h at 4°C. After centrifugation (2500 rpm × 5 min), the immunoprecipitate was washed with 1 ml PBS. Loading buffer, 20 μl, was added to the immunoprecipitated protein and boiled for 5 min followed by SDS-PAGE, and Western Blotting.

### Statistical analysis

Statistical analysis was carried out using SPSS 16.0 for Windows. Student’s *t*-test was used to analyze the results expressed as mean ± SD. Correlation analysis was used to analyze the relationship between miR-219 and SMC4. Differences were considered significant when the p values were less than 0.05.

## Results

### SMC4 is strongly upregulated in HCC

**Figure 1:**
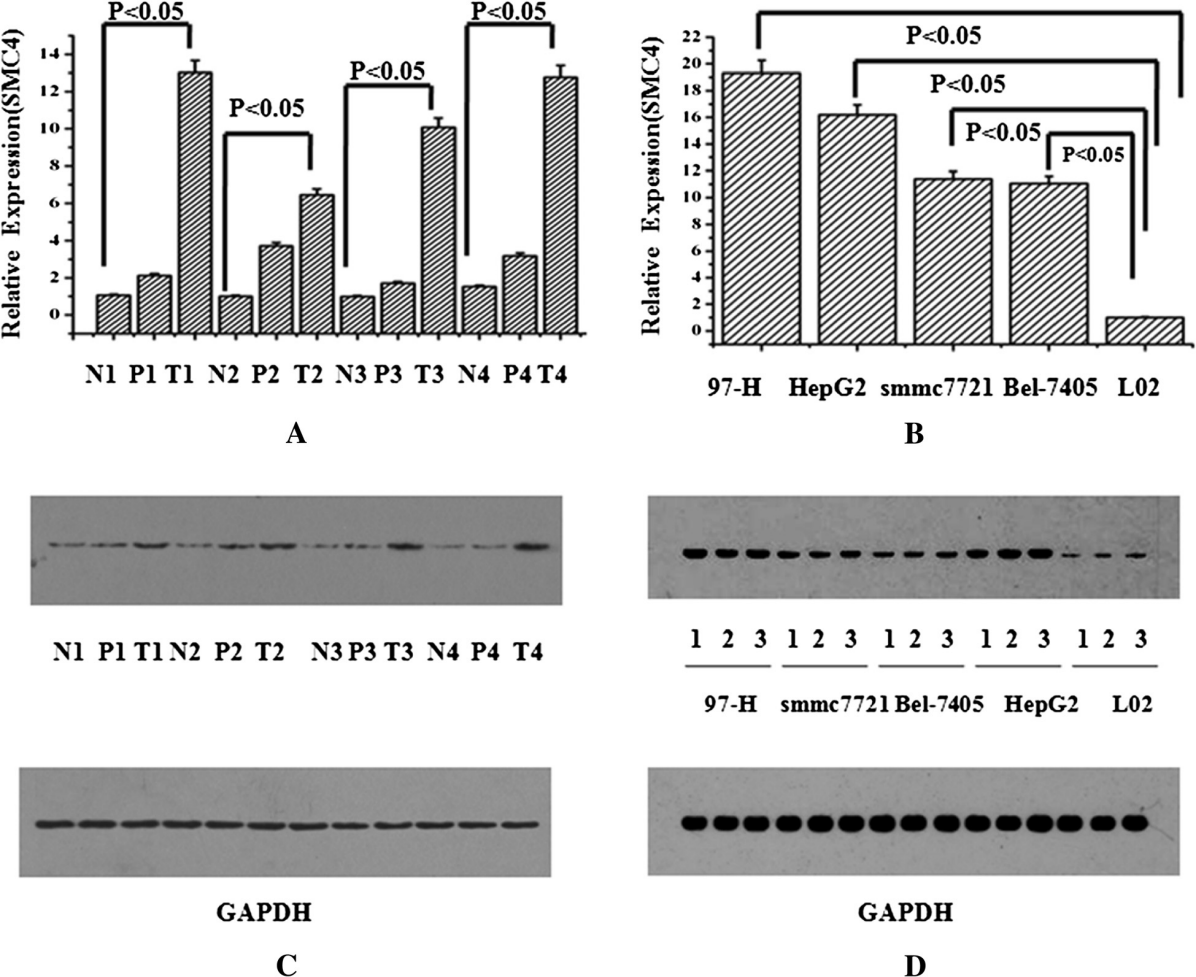
**Upregulation of SMC4 in HCC. (A)** Expression observed in 4 primary HCC samples (T) compared to paracancerous (P) and non-tumor (N) as determined by real-time quantitative PCR. **(B)** Expression observed in 4 HCC cell lines (97-H, HepG2, Bel-7405, and smmc-7721) compared to normal cells (L02) as determined by real-time quantitative PCR. **(C)** Expression observed in 4 primary HCC samples (T) compared to paracancerous (P) and non-tumor tissues (N) by Western Blotting. **(D)** Expression observed in 4 HCC cell lines (97-H, HepG2, Bel-7405, and smmc-7721) compared to normal cells (L02) by Western Blotting.

Real-time quantitative PCR and Western Blotting were performed to determine the expression of SMC4 in 4 HCC cell lines (97-H, HepG2, Bel-7405, and smmc-7721) and 4 primary HCC samples. The results showed that SMC4 was strongly upregulated in 4 HCC cell lines compared to normal cells (L02), and highly expressed in 4 primary HCC samples compared to paracancerous and non-tumor samples (Figure 1).

### SMC4 facilitates tumor cells proliferation, invasion and migration

**Figure 2:**
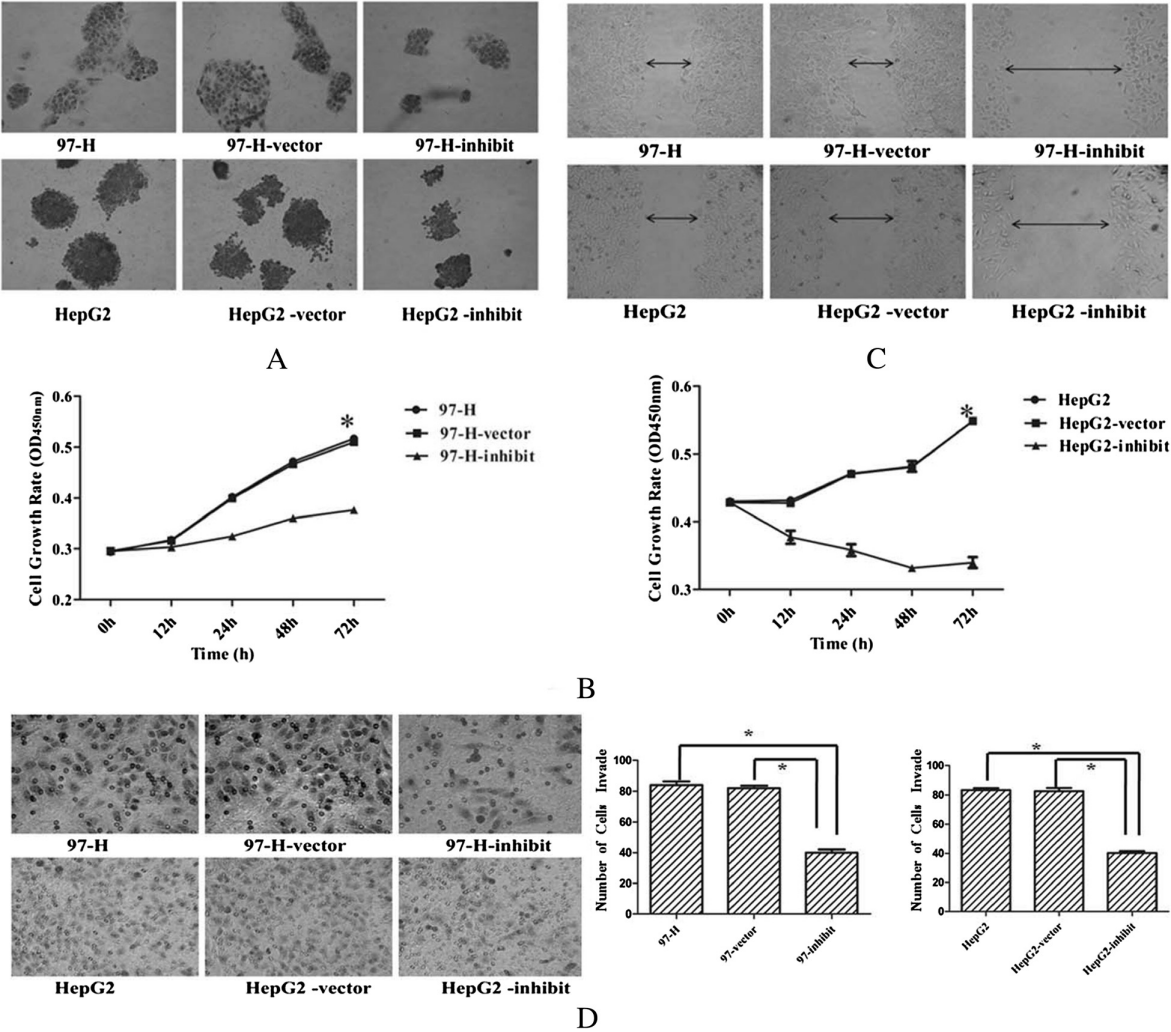
**SMC4 tumor-promoting effects (×400). (A)** Colony formation in soft agar after exposure to SMC4-homo-830. Representative images were taken at time 72 h after transfection. **(B)** Cell growth rates after exposure to SMC4-homo-830 as detected by WST-1 assay. **(C)** Wound-healing assays and cell motility after exposure to SMC4-homo-830. Representative images were taken at time 72 h after scratching. **(D)** SMC4-homo-830 decreased tumor cell invasion activity of 97-H and HepG2 cells. (*p < 0.05).

To explore the potential role of SMC4 in HCC, SMC4-homo-830 was cloned and stably transfected into cell lines (97-H and HepG2) and compared with controls. Cellular proliferation evaluated by soft agar colony formation assays showed that colony formation in soft agar was significantly inhibited in experimental groups compared with normal controls (Figure 2A). WST-1 assays also showed that the cell growth of 97-H and HepG2 cell lines were also significantly suppressed from 100% to 73.4% and 60.9% separately (Figure 2B). To further study the invasion and migration ability of SMC4, wound-healing and invasion assays were also performed. Wound-healing assays showed that cell migration rate was reduced compared with the empty vector-transfected and non-transfected groups (Figure 2C). Cell invasion assays also indicated that the number of invasive cells was significantly decreased (Figure 2D). However, statistical analysis demonstrated a significant difference in the association between experimental groups compared with normal controls (P < 0.05). Therefore, we conclude that SMC4 can promote the tumor cell proliferation, invasion and migration.

### Regulation of SMC4 Transcription by miR-219

**Figure 3:**
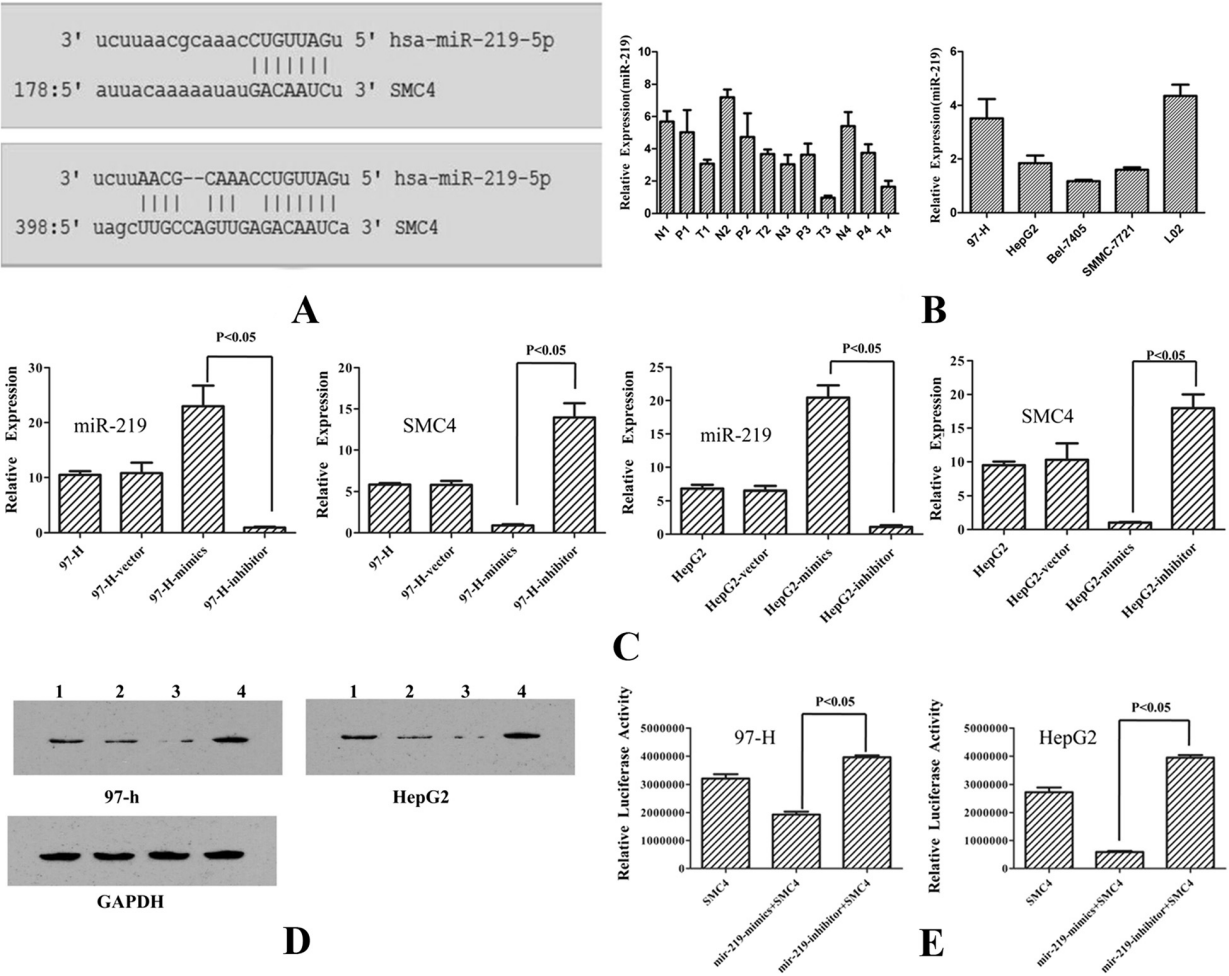
**SMC4 is a downstream target of miR-219. (A)** The predicted binding sequence of SMC4 and its binding site in the 3’-untranslated region (UTR) of SMC4 (sense) is presented for alignment. **(B)** Real-time quantitative PCR results showing miR-219 levels in HCC tissues and cell lines (T, HCC tissues; P, paracancerous tissues; N, non-tumor tissues). **(C)** Real-time quantitative PCR result showing that SMC4 upregulation by miR-219-inhibit, and also downregulation by overexpression of miR-219 in 97-H and HepG2 cell lines. **(D)** Western Blotting result showing that SMC4 upregulation by miR-219-inhibit, and also downregulation by overexpression of miR-219 in 97-H and HepG2 cell lines (1,97-H/HepG2; 2, 97-H/HepG2-vector; 3, 97-H/HepG2-minics; 4, 97-H/HepG2-inhibitor). **(E)** Luciferase reporter assays were used to confirm the interaction of miR-219 with SMC4. 3’-UTR of SMC4 containing the target binding site (sense) was cloned downstream of a firefly luciferase gene. The plasmids were transfected into empty vector and miR-219 stably expressing cells (97-H, HepG2).

Using five different bioinformatics software (TargetScan, miRDB, picTar, DIANA microRNA) to analysis 3’UTR of SMC4, we found that miR-219 has two binding sites (Figure 3A). Quantitative PCR analysis demonstrated that miR-219 was significantly downregulated, which was consistent with the data from HCC tissues and cell lines (Figure 3B). We evaluated the effect of miR-219 levels on SMC4 transcription. After the miR-219 mimics and miR-219 inhibitor were transfected into the 97-H and HepG2 cell lines, real-time quantitative PCR, and Western Blotting assays showed that miR-219 silencing caused a significant increase in SMC4 expression. MiR-219 overexpression caused a significantly decrease in the SMC4 expression in 97-H and HepG2 cell lines (Figure 3C and 3D). Luciferase reporter assays also confirmed a negative regulation relationship between miR-219 and SMC4 (Figure 3E).

### JAK2/Stat3 is the potential target of SMC4

**Figure 4:**
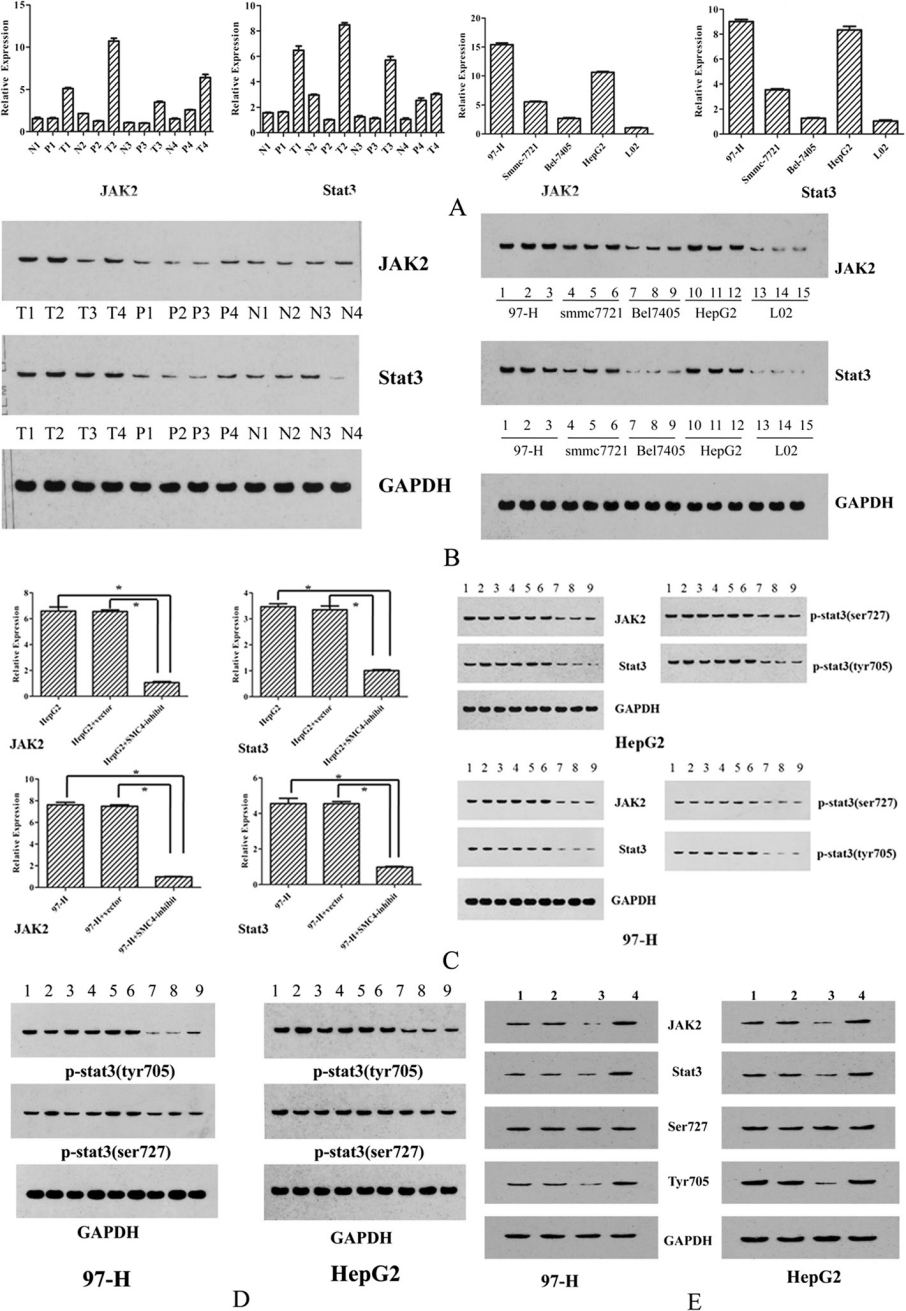
**JAK2/Stat3 is downstream target of SMC4. (A)** Real-time quantitative PCR results on JAK2/Stat3 expression in HCC tissues and cell lines (T, HCC tissues; P, paracancerous tissues; N, non-tumor tissues).** (B)** Western blotting results showing that JAK2/Stat3 was upregulation in HCC cell lines (T, HCC tissues; P, paracancerous tissues; N, non-tumor tissues). **(C)** Western Blotting (left) and real-time quantitative PCR (right) results showing JAK2/Stat3 downregulation by SMC4-inhibit in 97-H and HepG2 cell lines (1–3, 97-H/HepG2; 4–6, 97-H/HepG2 -vector; 7–9, 97-H/HepG2-inhibit). **(D)** Co-immunoprecipitation assays showing that p-Stat3 (Tyr705) protein expression after silencing of SMC4 in 97-H and HepG2 cell lines (1–3, 97-H/HepG2; 4–6, 97-H/HepG2 -vector; 7–9, 97-H/HepG2-inhibit). **(E)** Western Blotting results showing JAK2/Stat3 changed by after miR-219 modulation with mimics and inhibitors in 97-H and HepG2 cell lines, and phosphorylation status (1, 97-H/ HepG2; 2, 97-H/HepG2 -vector; 3, 97-H/HepG2-minics; 4, 97-H/ HepG2-inhibitor ). GAPDH was used as a control. (*p < 0.05).

By searching the potential targets of SMC4 with PIPs and Donime [20,21], JAK2/Stat3 was identified as the target of SMC4. Quantitative PCR and Western Blotting showed that JAK2/Stat3 was strongly upregulated at both RNA and protein levels (Figure 4A and Figure 4B). After the SMC4 inhibitor was transfected into the 97-H and HepG2 cell lines, real-time quantitative PCR and Western Blotting showed that SMC4 downregulated JAK2/Stat3 expression at both RNA and protein levels (Figure 4C). Co-immunoprecipitation was used to detect the phosphorylating modification of Stat3. The results indicated that p-Stat3 (Tyr705) protein expression decreased significantly after silencing of SMC4, and p- Stat3(Ser727)had no significant changes (Figure 4D). We conclude that the main changes in phosphorylation of p- Stat3 occurred at amino acid Tyr705, but not Ser727. After the miR-219 mimics, and miR-219 inhibitor were transfected into the 97-H and HepG2 cell lines, Western Blotting results showed that miR-219 silencing caused a significant increase in JAK2/Stat3 expression. miR-219 overexpression caused a significant decrease in the JAK2/Stat3 expression in 97-H and HepG2 cell lines. The results also indicated that p-Stat3 (Tyr705) protein expression changed significantly after modulation with miR-219, while p-Stat3(Ser727) had no significant changes (Figure 4E).

## Discussion

Structural maintenance of chromosomes (SMC) proteins are chromosomal ATPases, and are highly conserved from bacteria to humans. They play fundamental roles in many aspects of higher-order chromosome organization and dynamics [22]. SMC proteins act as global organizers and safeguards by directly and indirectly influencing chromosome structure and dynamics. They participate in a vast number of vital cellular processes ranging from cell division to gene regulation and DNA repair [23]. Individual eukaryotic organisms have at least six SMC family members that form three heterodimers in specific combinations: SMC1 and SMC3, SMC5 and SMC6, SMC2 and SMC4. SMC2 and SMC4 that are central components of condensin complexes [24]. SMC4 encodes a “structural maintenance of chromosomes” protein, and is a subunit of condensin. It has clear roles in chromosome condensation and mitosis, and is required for normal S phase progression, indicating a previously unrecognized role of Smc4p in the synchronous progression from G1 into S phase [25,26]. Research also suggests that SMC4 is a potential biomarker of the sensitivity of breast cancer cells to paclitaxel and the response to SAHA/paclitaxel combination treatment [27]. Previous studies have shown that the expression of SMC4 mRNA and protein was highly upregulated in HCC tissues and cell lines. Levels of SMC4 protein were significantly associated with tumor size, differentiation, TNM stage, and vascular invasion of primary liver cancer [17]. From data presented on these 72 cases published previously, the 4 specimens selected in the current study are representative of the others. In the present study, we also found that SMC4 was frequently upregulated in HCC cases and cell lines, consistent with our previous study. Therefore, we assumed that SMC4 played an important role in HCC development and invasion. In the current study, the effects of SMC4 on HCC development and invasion were investigated. One important task was to identify the upstream targets that regulate SMC4. Using five different bioinformatics software to analyze the 3’UTR of SMC4, miR-219 was found to be involved. A previous study also showed that miR-219 was potentially involved in gastric cancer progression and metastasis [28]. MiR-219 has been found to be significantly downregulated in HCC, and exert tumor-suppressive effects in hepatic carcinogenesis through negative regulation of GPC3 expression [15], which is consistent with our experimental results in HCC cell lines and tissues. Previous studies have shown that miR-219 inhibited cell proliferation [29,30]. There are several known pathways, including the GPC3 pathway by which the proliferation of HCC cells is regulated. GPC3 is a cell surface protein that has been implicated as a possible tumor marker for HCC [31]. In the current study, we found that SMC4 is also a target of miR-219 in the regulation of the proliferation and invasion of HCC cells. Correlation analysis showed that these two indexes had a trend of negative correlation (r = −0.394, p = 0.000). Quantitative PCR and Western Blotting analysis also demonstrated that miR-219 decreased SMC4 mRNA levels. In addition, inhibiting miR-219 expression increased SMC4 levels. Luciferase assays showed a negative regulatory relationship between miR-219 and SMC4. Furthermore, we identified the downstream target regulated by SMC4. JAK2/Stat3 was identified as the target of SMC4. JAK2, a member of the Janus (JAK) family of non-receptor protein tyrosine kinases, regulates signaling via multiple cytokine receptors [32,33]. Stat3, which is associated with oncogenesis, cell proliferation, angiogenesis, immune evasion, and apoptotic resistance, is constitutively activated in human HCC tissues, but not in normal human liver tissues [34-36]. One study showed that celecoxib decreased Stat3 phosphorylation by reducing Janus kinase (JAK2) phosphorylation, and caused apoptosis in HCC cells [37]. Peak Stat3 phosphorylation occurred within 15–60 min of exposure to cytokine. This constitutive activation of Stat3 is due to deregulation of protein tyrosine kinases or constitutive release of growth factors that activate Stat3 [38-40]. Activation of JAK2/ Stat3 signaling in solid tumors may represent a prognostic biomarker and therapeutic target [41].

In the present study, quantitative PCR and Western Blotting analysis demonstrated that JAK2/ Stat3 was significantly upregulated in HCC tissues and cell lines. Quantitative PCR and Western blotting analysis also demonstrated that SMC4 increased the expression of JAK2/ Stat3 at mRNA and protein levels. In addition, inhibition of SMC4 expression decreased JAK2/Stat3 expression. Phosphorylation of Stat3 has been shown to occur both at the tyrosine 705 (Y705) and at the serine 727 (S727) residues in the cytoplasmic tail [42]. Co-immunoprecipitation in the current study showed that the main change of phosphorylation of p-Stat3 occurred at amino acid Y705, but not at S727. Western Blotting analysis also showed that miR-219 resulted in significant changes in JAK2/Stat3 expression. p-Stat3 (Tyr705) protein expression also changed significantly after modulation with miR-219, but the p-Stat3(Ser727) had no significant changes.

## Conclusions

In summary, we have identified a novel miR-219/SMC4/JAK2/Stat3 signaling pathway whose function may contribute to the development of HCC. Our findings provide new insight into SMC4 function and the mechanisms of growth and invasion in HCC.

## Abbreviations

SMC4: Structural maintenance of chromosome 4; HCC: Hepatocellular Carcinoma.

## Competing interests

The authors declare that they have no competing interests.

## Authors’ contributions

PC conceived and designed the experiments. BZ, HXC, DW, YK and GYL performed the experiments. BZ, HXC and JX analyzed the data. BZ wrote the paper. PC supervised the whole experimental work and revised the manuscript. All authors read and approved the manuscript.

## Supplementary Material

Additional file 1 Recombinant plasmid construct report. Click here for file
