# Correction to: A novel miR-219-SMC4-JAK2/Stat3 regulatory pathway in human hepatocellular carcinoma

**DOI:** 10.1186/s13046-021-02028-z

**Published:** 2021-07-05

**Authors:** Bo Zhou, Hongxu Chen, Dong Wei, Yi Kuang, Xiaobiao Zhao, Guangyao Li, Jun Xie, Ping Chen

**Affiliations:** Department of Hepatobiliary Surgery, Daping Hospital and Research Institute of Surgery, The Third Military Medical University, Chongqing, China

**Correction to: J Exp Clin Cancer Res 33, 55 (2014)**

**https://doi.org/10.1186/1756-9966-33-55**

Following publication of the original article [1], the authors identified minor errors in image-typesetting in Fig. 2; specifically, in Fig. 2D, the ‘97-h’ panel (top left) has been replaced by the correct image.

**Fig. 2:**
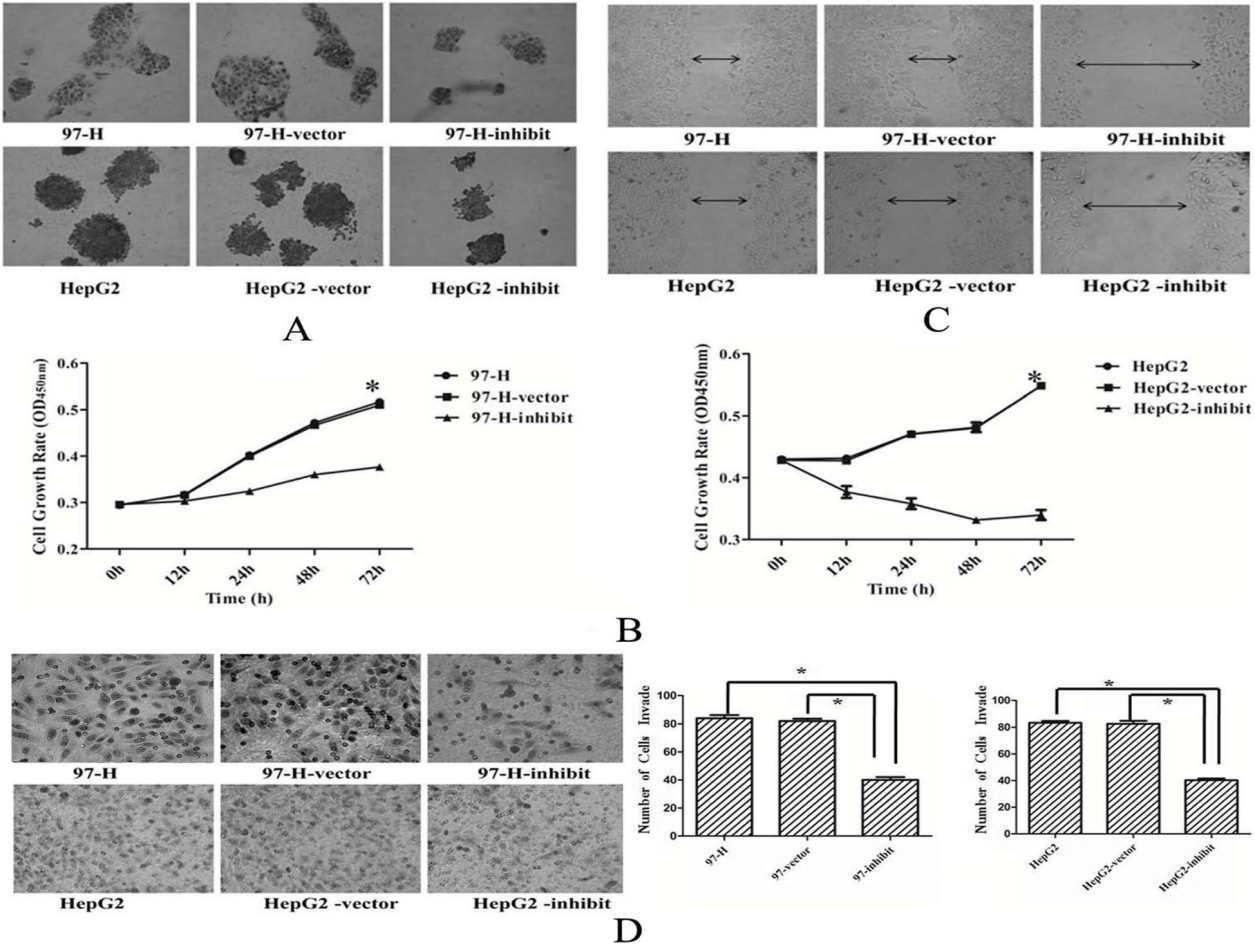
SMC4 tumor-promoting effects (× 400). **A** Colony formation in soft agar after exposure to SMC4-homo-830. Representative images were taken at time 72 h after transfection. **B** Cell growth rates after exposure to SMC4-homo-830 as detected by WST-1 assay. **C** Wound-healing assays and cell motility after exposure to SMC4-homo-830. Representative images were taken at time 72 h after scratching. **D** SMC4-homo-830 decreased tumor cell invasion activity of 97-H and HepG2 cells. (*p < 0.05)

The corrected figure is provided here. The corrections do not have any effect on the results or conclusions of the paper. The original article has been corrected.
