# Supplementary material for: A novel miR-219-SMC4-JAK2/Stat3 regulatory pathway in human hepatocellular carcinoma
Source: J Exp Clin Cancer Res. 2014 Jun 30;33(1):55. doi: 10.1186/1756-9966-33-55 (PMC4096530; doi:10.1186/1756-9966-33-55)
Supplement: Additional file 1 — Recombinant plasmid construct report. [file 1756-9966-33-55-S1.doc]

**Supply 1:** Recombinant plasmid construct report. The insert sequence information as follows:

| Gene/insert name: | 3'UTR of SMC4 |
| --- | --- |
| Official Symbol: | SMC4 |
| Gene ID: | 10051 |
| Gene type: | protein coding |
| Organism: | Homo sapiens |
| RefSeq: | NM_005496.3 |
| 5'Primers: | actttatgctgaagattcttcaag |
| 3'Primers: | tttccgatcttgctttctttaaatc |
| Insert size: | 1153 bp |
| Insert sequence: | actttatgctgaagattcttcaagttgattcagtgtattactgatttttttctatttgtaaaggattatgagttgtataaaatacatactccctaaactagatcatgaaactggtttctgttttatgcagttgtcatttgtaaagtctaataaaatattctctataattgcttctagattacaaaaatatgacaatcttgtaagtagcagactatggagaaaaatgagttacctggagggtcaggtaacttgccaaactaaaaagtatgttagttgaggcaaagtcctaagcaaggttgtgctatcaaggctcagcataccttcgtgggcctttgatttaccaacactggaaatgcctgccaactaatcttggatagattctttaaggcattccacttagcttgccagttgagacaatcaccacagttattacccaaatactatgaacatatttttgtaaaccagtcattctgaattatagtgatgagaatttaaatatatgcttttctagaatttgatgtttgaccatttatgacttaattaccagagagccagtaaattaggacagtgtttcaacaagcctaggctatctcgtaagttgaaaaatatcccactatagttgcttcatgagtatgaagtaagatggcctctgatttacactggttcaatttacaaattttcaactttatgataggtttatccgggtactaaatgcatttcaacttgatagtttcaacttatgataggtttaccaggatgtagtcccactgttgaggagcatctatttaggggttaattactttagtaataagtggaaagtaagataccttgagtaatgtttgcctataaaattgtcagcgtatttttacactattggctcaagaatgttataatgctaagggacataagttggcaaccacttggtttttggaaggactttcggtattgtattagaagtctgccctagctgttaaatttctgggtatttatcctaaggaattaattaaagagttaattgttcctttcttcagtgggccattgttttagatatttaaaaaatccaacagtttctatcataatgtaactgtaaaaatgtaaacacattattagcatggacttttaaataaagatttaaagaaagcaagatcggaaa |
